# Association of MOCA Cognitive Domains and Serum Biomarkers With Anxiety Disorders in Elderly Men With Cognitive Impairment: A Cross-Sectional Analysis

**DOI:** 10.31083/RN48908

**Published:** 2026-04-21

**Authors:** Xiao Chen, Tong Li, Jiawei Zhang, Wei Zhou, Jiayun Dai

**Affiliations:** ^1^Geriatrics Ward, Jiangsu Rongjun Hospital, 214062 Wuxi, Jiangsu, China

**Keywords:** aged, cognitive dysfunction, anxiety disorders, Mental Status and Dementia Tests, biomarkers, cross-sectional studies

## Abstract

**Background:**

Anxiety symptoms in elderly patients with cognitive impairment (CI) often reflect shared neurobiological processes rather than distinct psychiatric disorders. Current diagnostic approaches lack objective biomarkers for early identification. This study investigated the association between serum biomarkers and anxiety disorder status in elderly men with CI and to evaluate the exploratory discriminative ability of cognitive domains and biomarker profiles in differentiating CI patients with and without comorbid anxiety.

**Methods:**

This cross-sectional retrospective study analyzed 86 elderly male CI patients (Group A: CI alone, n = 41; Group B: CI with anxiety, n = 45) at Jiangsu Rongjun Hospital (June–December, 2024). Anxiety disorder diagnosis was established through structured clinical interviews based on the Diagnostic and Statistical Manual of Mental Disorders, Fifth Edition (DSM-5) criteria conducted by two independent psychiatrists, with the Hamilton Anxiety Scale (HAMA) serving as an initial severity screening instrument. The Montreal Cognitive Assessment (MOCA) was used to evaluate cognitive function. Enzyme-linked immunosorbent assay (ELISA) was used to measure serum Tau protein (Tau), β-amyloid (Aβ), visinin-like protein 1 (VILIP-1), malondialdehyde (MDA), tumor necrosis factor-α (TNF-α), and interleukin-6 (IL-6); reverse transcription-polymerase chain reaction (RT-PCR) quantified microRNA-34c (*MiR-34c*). Patients with acute inflammation (C-reactive protein [CRP] >10 mg/L) were excluded. Bonferroni correction was used to address multiple comparisons across 25 simultaneous tests, and multivariate regression analysis was controlled for demographic and clinical confounders. Receiver operating characteristic (ROC) analysis was used to determine the discriminative ability.

**Results:**

Group B showed worse cognitive performance across the MOCA domains, with attention (area under the curve [AUC] = 0.738) and delayed recall (AUC = 0.742) demonstrating the strongest discriminative ability. Biomarker analysis revealed elevated Tau (AUC = 0.957), MDA (AUC = 0.941), and VILIP-1 (AUC = 0.914) in anxiety patients. Within-group analyses showed that anxiety severity correlated negatively with MiR-34c and positively with Tau, Aβ, MDA, IL-6, and VILIP-1. Under the Bonferroni-adjusted threshold (*p* < 0.002), only MDA in Group B (r = 0.478, *p* = 0.001) and MiR-34c in Group B (r = –0.523, *p* < 0.001) remained significant. Multivariate analysis identified these factors as independently associated with the outcome after controlling for demographics and comorbidities. However, given the substantial baseline imbalances between the groups, these associations should be interpreted with caution.

**Conclusion:**

Combined cognitive assessment (attention, delayed recall) and serum biomarkers (Tau, MDA, VILIP-1, MiR-34c) demonstrate promising discriminative ability for identifying anxiety in elderly male patients with CI. These findings are exploratory and derived from a single-center cohort of retired male military veterans with pronounced baseline group imbalances, which substantially limits generalizability to the broader elderly CI population. The identified markers may reflect shared neuroinflammatory and oxidative stress pathways underlying both cognitive and emotional dysfunction, warranting further investigation as potential targets for integrated therapeutic approaches. Validation in prospective, multicenter, sex-inclusive cohorts with balanced comparison groups is essential before any clinical application can be considered.

## 1. Introduction

Anxiety disorders are prevalent among the elderly, though reported prevalence rates vary considerably depending on the population studied and the diagnostic criteria employed. In community-dwelling elderly populations, systematic reviews and meta-analyses have reported prevalence estimates ranging from 3.2% to 14.2%, while rates are substantially higher in clinical and institutionalized settings, where estimates may exceed 20%–28% in populations with comorbid chronic medical conditions [1, 2]. While prior research has predominantly examined younger populations, evidence suggests that anxiety disorders in elderly individuals cause greater psychological and physical distress, severely impacting their social engagement and mental well-being [3]. Therefore, early identification and intervention for anxiety disorders have become critical in clinical management.

The relationship between cognitive impairment (CI) and anxiety disorders in elderly populations is complex and bidirectional [4, 5]. Neurobiologically, both conditions share common pathophysiological mechanisms, including dysfunction in prefrontal-limbic circuits, dysregulation of neurotransmitter systems (particularly serotonin, gamma-aminobutyric acid (GABA), and dopamine), and alterations in the hypothalamic-pituitary-adrenal axis [6, 7, 8, 9]. In elderly patients with CI, structural brain changes in regions such as the amygdala, hippocampus, and prefrontal cortex can precipitate anxiety symptoms, while chronic anxiety may accelerate cognitive decline through sustained stress responses and inflammatory cascades [10, 11].

The temporal relationship between CI and anxiety is particularly relevant in clinical practice. While anxiety can manifest as a reaction to cognitive decline awareness, it can also represent an early neuropsychiatric symptom of underlying neurodegenerative processes [12]. Studies have shown that anxiety disorders in patients with mild cognitive impairment increase the risk of progression to dementia, suggesting shared pathological mechanisms [13, 14]. Furthermore, the cognitive domains most affected by anxiety disorders—attention, executive function, and memory—overlap significantly with those impaired in CI, creating a synergistic effect that compounds functional disability [14]. Importantly, in the context of neurodegenerative diseases, anxiety symptoms may constitute behavioral and psychological symptoms of dementia (BPSD) rather than primary anxiety disorders, and distinguishing between these entities remains a significant clinical challenge [13, 14].

Current diagnostic approaches, which rely on medical history and psychological scales, are subjective and often lead to misdiagnosis or delayed detection. Elderly patients are frequently diagnosed only after the manifestation of severe symptoms [5]. This challenge is compounded by the fact that anxiety symptoms in elderly patients with CI may be attributed to cognitive decline itself, leading to under-recognition and under-treatment [15, 16]. Although extensive research has explored the neurophysiological mechanisms and risk factors associated with anxiety disorders, there is limited research on early screening using serum biomarkers [6]. Thus, the development of objective assessment methods to identify high-risk individuals early is imperative.

Recent advancements in biomarker detection technologies have highlighted the potential of serum markers in the diagnosis of neurological disorders [7, 10]. Tau protein (Tau) and β-amyloid (Aβ) are established indicators of CI and have been implicated in anxiety pathways through their effects on synaptic plasticity and neuroinflammation [17, 18]. Visinin-like protein 1 (VILIP-1) modulates neural signaling and is implicated in CI pathology and stress response mechanisms [19]. Inflammatory markers such as tumor necrosis factor-α (TNF-α) and interleukin-6 (IL-6) not only contribute to neurodegeneration but also directly influence mood regulation through their effects on serotonin metabolism and hypothalamic-pituitary-adrenal (HPA) axis function [20, 21]. Oxidative stress markers, such as malondialdehyde (MDA), reflect cellular damage that affects both the cognitive and emotional processing regions of the brain [22]. MicroRNA-34c (MiR-34c), a key regulator, is associated with anxiety behaviors and modulates anxiety-related pathways by regulating serotonin receptors and stress-response genes [23].

This study sought to analyze clinical data and serum samples from elderly male CI patients to identify biomarkers associated with anxiety disorders, offering novel perspectives for the characterization and future investigation of early detection and intervention in elderly men.

## 2. Materials and Methods

### 2.1 General Information

This study included elderly male CI patients undergoing short-term rehabilitation between June and December 2024. A priori power analysis indicated that a sample size of 80 participants (40 per group) would provide 80% power to detect a medium effect size (Cohen’s d = 0.65) with α = 0.05. A total of 86 participants were ultimately enrolled (Group A: n = 41; Group B: n = 45), exceeding the minimum required sample size.

Participants met the following criteria: (1) Age ≥60 years; (2) Ability to complete cognitive and anxiety assessments with guidance; (3) CI diagnosis based on Montreal Cognitive Assessment (MOCA) score <26 determined during the admission assessment period; (4) Informed consent from patients or families.

Exclusion criteria included: (1) Comorbid malignancies or terminal illnesses; (2) History of anxiety, depression, or other psychiatric disorders prior to CI onset; (3) History of traumatic brain injury, hemorrhagic stroke, or Parkinson’s syndrome; (4) Acute inflammatory conditions (C-reactive protein [CRP] >10 mg/L, fever >38 °C, active infection within 2 weeks).

### 2.2 Participant Enrollment and Diagnostic Characterization

Patients were consecutively enrolled upon admission for short-term rehabilitation. Cognitive impairment was identified using the MOCA assessment conducted during the admission evaluation. The enrolled population comprised patients whose cognitive symptoms were identified either during the current admission or through prior clinical records; however, the retrospective nature of the study limited the precise determination of disease duration for all participants. All participants underwent the anxiety assessment protocol described below at enrollment.

### 2.3 Anxiety Disorder Assessment

The Hamilton Anxiety Scale (HAMA) [10] was used as an initial screening instrument for anxiety symptom severity. The HAMA is a clinician-administered rating scale comprising 14 items, each scored on a 0–4 scale. A total score >14 was used as the threshold to identify patients who required further diagnostic evaluation. It is important to note that the HAMA is designed as a severity measure rather than a standalone diagnostic tool; accordingly, the final anxiety disorder diagnosis was established through structured clinical interviews based on the Diagnostic and Statistical Manual of Mental Disorders, Fifth Edition (DSM-5) criteria, conducted independently by two board-certified psychiatrists. Inter-rater reliability was assessed using κ = 0.89. Clinical consensus was required for the final diagnosis of anxiety disorder.

### 2.4 Clinical Data Collection

A structured questionnaire was used to collect data on age, marital status, education, income, family structure, body mass index (BMI), cardiac function, smoking, alcohol use, trauma history, and medical history (hypertension, diabetes, coronary heart disease, and ischemic stroke). Fasting venous blood samples were collected after 12 h to measure blood cell counts, total bilirubin (TBIL), alanine aminotransferase (ALT), aspartate aminotransferase (AST), total cholesterol (TC), triglycerides (TG), low-density lipoprotein cholesterol (LDL-C), urea nitrogen (UREA), creatinine (CRE), uric acid (UA), fasting blood glucose (FBG), CRP, and homocysteine (HCY) levels.

### 2.5 Serum Biomarker Detection

Serum concentrations of Tau, Aβ, VILIP-1, superoxide dismutase (SOD), MDA, TNF-α, and IL-6 were measured using commercially available ELISA kits according to the manufacturers’ instructions. The VILIP-1 ELISA kit was purchased from Roche Diagnostics (Shanghai, China; Cat. No. 11776). Procedures adhered to manufacturer protocols: (1) collecting 3 mL of fasting venous blood, isolating serum, and storing at –80 °C; (2) coating 96-well plates with capture antibodies, adding samples and standards, and incubating; (3) washing, adding detection antibodies and enzyme conjugates, and incubating further; and (4) adding substrate and measuring absorbance at 450 nm. All assays were performed in duplicate, and the intra-assay and inter-assay coefficients of variation were <5% and <10%, respectively. All laboratory personnel performing enzyme-linked immunosorbent assay (ELISA) were blinded to the clinical grouping of the participants throughout the analytical process.

### 2.6 Serum MiR-34c Level Detection

Quantification of *MiR-34c* in serum was performed using reverse transcription-polymerase chain reaction (RT-PCR) kits were obtained from Tianenze Biotech Co., Ltd. (Cat. No. TNZ-M3421; Shanghai, China), adhering to the manufacturer’s protocol: (1) drawing 3 mL of venous blood after fasting, separating serum, and preserving at –80 °C; (2) isolating total RNA, measuring concentration and purity via absorbance at 260 nm and 280 nm; (3) Conducting RT-PCR following polyadenylation of miRNA, cDNA generation, and 40 cycles of amplification (95 °C for 15 s, 60 °C for 1 min). Relative *MiR-34c* expression was determined using the ΔCt approach with *U6* snRNA as an internal control. The laboratory personnel performing RT-PCR were blinded to the participant group assignments.

### 2.7 Statistical Analysis

Analysis of data utilized SPSS 22.0 software (IBM Corp., Armonk, NY, USA). The normality of continuous variables was assessed using the Shapiro-Wilk test. Continuous measurements are presented as mean ± standard deviation, with comparisons performed using independent-samples *t*-tests for normally distributed variables or Mann-Whitney U tests (reported as z-values) for variables violating normality assumptions. Categorical data were reported as percentages and evaluated using chi-square tests. The relationships between anxiety levels and various parameters within each group were examined using Pearson’s correlation for normally distributed data or Spearman’s correlation for non-normally distributed data to avoid confounding by group differences. The choice between the Pearson and Spearman methods for each analysis is specified in the corresponding table footnotes. Bonferroni correction was applied for multiple comparisons (adjusted α = 0.002 for 25 simultaneous comparisons encompassing cognitive domains, blood biochemistry parameters, and serum biomarkers). Multivariate logistic regression analysis was performed to identify the factors independently associated with anxiety disorders, adjusting for age, education, smoking, and comorbidities. Variance inflation factors (VIF) were calculated to assess multicollinearity among the predictor variables. Receiver operating characteristic (ROC) analysis was performed to evaluate the discriminative ability of individual cognitive domains and serum biomarkers; however, it should be noted that univariate ROC analyses do not account for confounding variables and should be interpreted as exploratory assessments of discrimination performance within this specific cohort rather than as evidence of diagnostic utility. Statistical significance was set at *p* < 0.05 for primary analyses and *p* < 0.002 for multiple comparisons. All *p* values are reported to three decimal places, with values less than 0.001 reported as *p* < 0.001.

## 3. Results

### 3.1 Baseline Demographic and Clinical Characteristics

Prior to presenting the outcome comparisons, the baseline demographic and clinical characteristics of the study population were described. A total of 86 elderly male patients with CI were included, with 41 in Group A (CI alone) and 45 in Group B (CI with comorbid anxiety disorder). The exclusively male composition of this cohort reflects the institutional setting: Jiangsu Rongjun Hospital primarily serves retired military veterans, who are overwhelmingly male in the current Chinese elderly population. During the study period, too few female CI patients met the inclusion criteria to permit meaningful sex-stratified analysis; the implications of this restriction are addressed in the Limitations section. As presented in Table 1, substantial baseline differences were observed between groups. Group B had a significantly higher proportion of patients aged ≥70 years (66.67% vs. 24.39%, *p* < 0.001), lower educational attainment (82.22% with ≤9 years of education vs. 68.29%, *p* = 0.022), and a higher prevalence of comorbid conditions, including hypertension (68.89% vs. 48.78%, *p* = 0.006), diabetes (51.11% vs. 21.95%, *p* < 0.001), coronary heart disease (26.67% vs. 14.63%, *p* = 0.036), and ischemic stroke (15.56% vs. 2.44%, *p* < 0.001). Higher rates of New York Heart Association (NYHA) class ≥2 cardiac dysfunction (68.89% vs. 53.66%, *p* = 0.029), smoking (64.44% vs. 48.78%, *p* = 0.032), and trauma history (53.33% vs. 34.15%, *p* = 0.007) were also observed in Group B. No significant differences were found in marital status, income, family structure, BMI, and alcohol use. These baseline imbalances are acknowledged as significant limitations and were addressed through multivariate adjustment, although residual confounding cannot be excluded (Table 1).

**Table 1.** **Comparison of baseline demographic and clinical characteristics between patients with CI without anxiety (Group A) and patients with CI with comorbid anxiety disorder (Group B) (case count and percentages)**.

Project		Group A (*n* = 41)	Group B (*n* = 45)	χ ^2^	*p*
Age				38.585	<0.001*
	≥70 years old [case count (%)]	10 (24.39)	30 (66.67)		
	<70 years old [case count (%)]	31 (75.61)	15 (33.33)		
Marriage				0.700	0.403
	Normal [case count (%)]	40 (97.56)	43 (95.56)		
	Widowed and divorced [case count (%)]	1 (2.44)	2 (4.44)		
Education				5.281	0.022*
	Be educated ≤9 years [case count (%)]	28 (68.29)	37 (82.22)		
	Be educated >9 years [case count (%)]	13 (31.71)	8 (17.78)		
Income				0.023	0.879
	<3000 CNY per month [case count (%)]	13 (31.71)	14 (31.11)		
	≥3000 CNY per month [case count (%)]	28 (68.29)	31 (68.89)		
Family structure				2.033	0.154
	Large family type [case count (%)]	25 (60.98)	23 (51.11)		
	Intermediate type [case count (%)]	16 (39.02)	22 (48.89)		
BMI				0.523	0.469
	<24 kg/m^2^ [case count (%)]	15 (36.59)	19 (42.22)		
	≥24 kg/m^2^ [case count (%)]	26 (63.41)	26 (57.78)		
Heart function				4.775	0.029*
	NYHA classification <2 [case count (%)]	19 (46.34)	14 (31.11)		
	NYHA classification ≥2 [case count (%)]	22 (53.66)	31 (68.89)		
	Smoking habit [case count (%)]	20 (48.78)	29 (64.44)	4.596	0.032*
	Drinking habit [case count (%)]	7 (17.07)	10 (22.22)	0.798	0.372
	History of trauma [case count (%)]	14 (34.15)	24 (53.33)	7.393	0.007*
	Combined hypertension [case count (%)]	20 (48.78)	31 (68.89)	7.487	0.006*
	Combined diabetes [case count (%)]	9 (21.95)	23 (51.11)	18.525	<0.001*
	Combined coronary heart disease [case count (%)]	6 (14.63)	12 (26.67)	4.389	0.036*
	Combined ischemic stroke [case count (%)]	1 (2.44)	7 (15.56)	13.473	<0.001*

CI, cognitive impairment; BMI, body mass index. NYHA, New York Heart Association. *p < 0.05. At the time of the study (2024), 1 USD ≈ 7.1 CNY (Chinese Yuan).

### 3.2 Cognitive Function and Anxiety Levels in CI Patients

Group A demonstrated higher MOCA total scores and better performance in visuospatial/executive function, attention, language, abstraction, and delayed recall than Group B. Naming and orientation showed no significant differences. HAMA scores confirmed elevated anxiety in Group B (Table 2; *p* < 0.05).

**Table 2.** **Comparison of cognitive function and anxiety levels between the two groups of patients (points, mean ± standard deviation)**.

Project	Group A (*n* = 41)	Group B (*n* = 45)	*t/z*	*p*
Total score of MOCA	24.15 ± 0.94	19.73 ± 2.38	–7.661	<0.001*
Visuospatial and executive abilities	3.49 ± 0.84	2.76 ± 1.07	–3.451	<0.001*
Naming	2.71 ± 0.56	2.73 ± 0.54	–0.299	0.765
Attention	5.02 ± 0.94	3.84 ± 1.33	–7.922	<0.001*
Language	2.51 ± 0.55	1.93 ± 0.75	–3.611	<0.001*
Abstraction	1.56 ± 0.50	1.00 ± 0.71	–3.761	<0.001*
Delayed recall	2.32 ± 1.19	1.09 ± 0.95	–4.631	<0.001*
Orientation	5.83 ± 0.44	5.67 ± 0.56	–1.570	0.117
HAMA	5.24 ± 1.18	18.67 ± 2.63	–8.091	<0.001*

MOCA, Montreal Cognitive Assessment; HAMA, Hamilton Anxiety Scale. *p < 0.05.

### 3.3 Correlation Between Cognitive Function and Anxiety Levels

Within-group correlation analysis revealed significant negative correlations between anxiety levels and MOCA total scores (Group A: r = –0.423, *p* < 0.01; Group B: r = –0.456, *p* < 0.01), attention (Group A: r = –0.389, *p* < 0.05; Group B: r = –0.421, *p* < 0.01), and delayed recall (Group A: r = –0.345, *p* < 0.05; Group B: r = –0.398, *p* < 0.01) in both groups, suggesting that cognitive performance is associated with anxiety severity independent of group membership (Table 3).

**Table 3.** **Within-group correlation analysis between cognitive function level and anxiety degree (Pearson’s correlation coefficients)**.

Project		Total score of MOCA	Visuospatial and executive	Attention	Language	Abstraction	Delayed recall
Group A HAMA							
	r	–0.423	–0.287	–0.389	–0.245	–0.298	–0.345
	*p*	0.006*	0.068	0.012*	0.121	0.059	0.028*
Group B HAMA							
	r	–0.456	–0.312	–0.421	–0.289	–0.334	–0.398
	*p*	0.002*	0.037*	0.005*	0.055	0.024*	0.007*

*Significant at *p* < 0.05. None of the within-group correlations survived Bonferroni correction (*p* < 0.002).

### 3.4 ROC Curve Analysis of Cognitive Function for Anxiety Disorder Discrimination

**Fig. 1.**
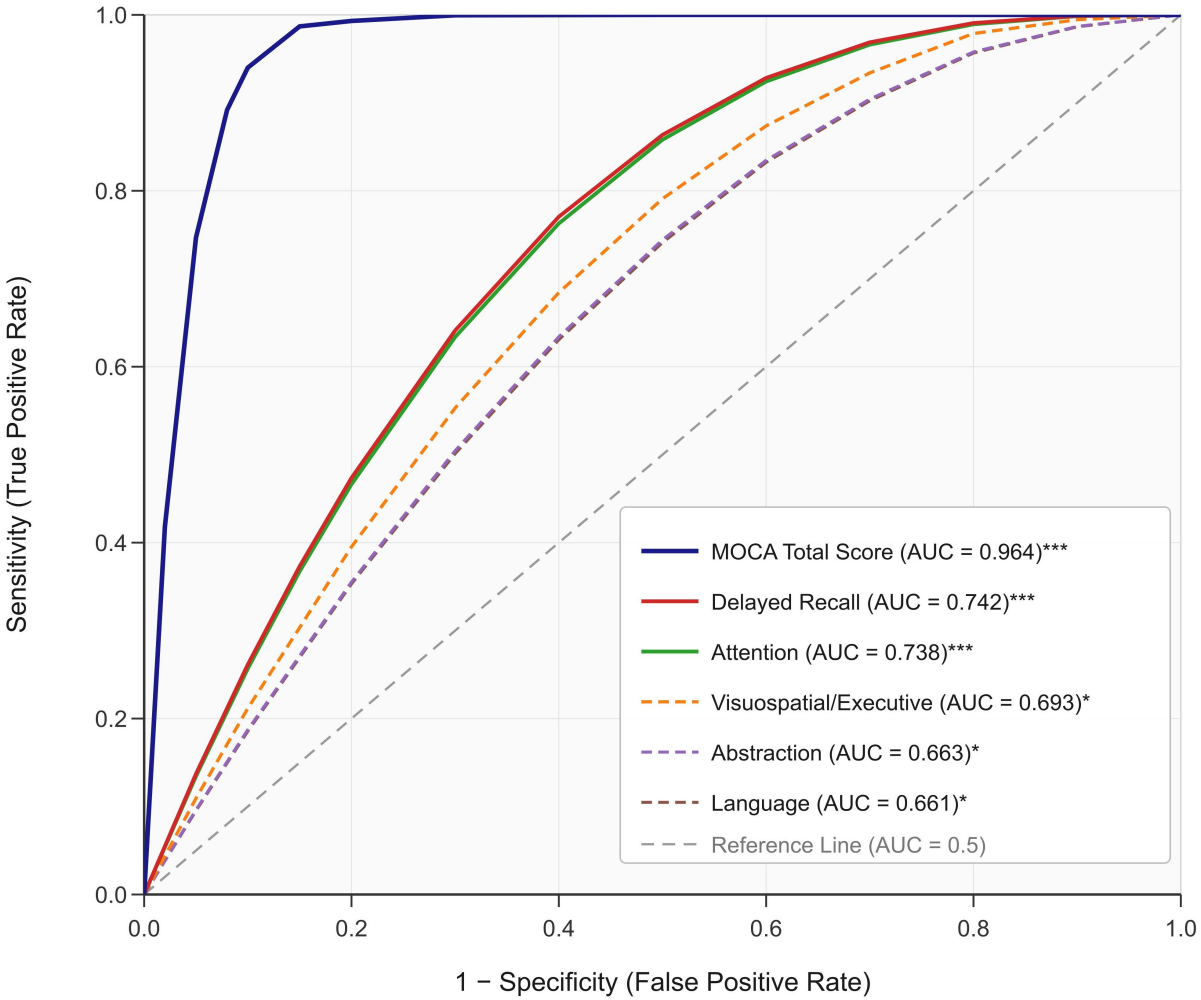
**ROC curve of cognitive function assessment factors for discriminating anxiety disorder status**. Receiver operating characteristic curves are shown for the MOCA total score and six cognitive subdomains. The diagonal dashed reference line represents chance discrimination (AUC = 0.5). Solid lines denote measures with AUC >0.70; dashed lines denote measures with AUC <0.70. The MOCA total score demonstrates excellent discrimination (AUC = 0.964, 95% CI: 0.921–1.000), while delayed recall (AUC = 0.742, 95% CI: 0.634–0.850) and attention (AUC = 0.738, 95% CI: 0.628–0.847) show good discriminative ability. AUC, area under the curve. ***p < 0.001; *p < 0.05. Optimal cut-off points were determined using Youden’s index. ROC, receiver operating characteristic.

ROC analysis evaluated the discriminative ability of cognitive measures in differentiating between patients with CI with and without comorbid anxiety disorder. ROC analysis indicated that the MOCA total scores, visuospatial/executive function, attention, language, abstraction, and delayed recall had area under the curve (AUC) values of 0.964, 0.693, 0.738, 0.661, 0.663, and 0.742, respectively, supporting their discriminative utility. MOCA total scores showed excellent discrimination (AUC = 0.964, 95% CI: 0.921–1.000, *p* < 0.001), whereas attention (AUC = 0.738, 95% CI: 0.628–0.847, *p* < 0.001) and delayed recall (AUC = 0.742, 95% CI: 0.634–0.850, *p* < 0.001) demonstrated good discriminative ability (Fig. 1). It should be noted that the exceptionally high AUC for the MOCA total score likely reflects the inherent relationship between overall cognitive severity and clinical group assignment, and these univariate ROC values do not account for confounding variables.

### 3.5 Blood Biochemical and Serum Biomarker Levels in CI Patients

Group A showed higher lymphocyte count (LYM) and MiR-34c levels, while Group B had elevated UREA, HCY, Tau, Aβ, MDA, TNF-α, IL-6, and VILIP-1 levels, suggesting that these markers are associated with CI and anxiety disorders (Table 4). The specific statistical test used for each variable (*t*-test or Mann-Whitney U test) is indicated in the table footnote based on the distribution of each variable, as assessed by the Shapiro-Wilk test.

**Table 4.** **Comparison of blood biochemistry and serum biomarker levels between two groups of patients (mean ± standard deviation)**.

Project	Group A (*n* = 41)	Group B (*n* = 45)	*t/z*	*p*
Hemoglobin [Hb] (g/L)	150.05 ± 9.62	145.87 ± 13.01	–1.138	0.255
Lymphocyte count [LYM] (10^9^/L)	1.86 ± 0.49	1.62 ± 0.44	2.430	0.017*
Platelet count [PLT] (10^9^/L)	197.71 ± 46.18	192.42 ± 57.78	0.466	0.643
TBIL (µmol/L)	14.98 ± 6.84	15.48 ± 6.32	–0.553	0.580
ALT (U/L)	21.53 ± 9.78	22.53 ± 10.34	–0.368	0.713
AST (U/L)	20.41 ± 5.46	23.20 ± 10.73	–1.077	0.282
TC (mmol/L)	5.57 ± 1.40	5.76 ± 1.14	–0.601	0.548
TG (mmol/L)	1.64 ± 1.25	1.26 ± 0.68	–1.807	0.071
LDL-C (mmol/L)	2.85 ± 0.68	2.86 ± 0.76	–0.007	0.994
UREA (mmol/L)	6.20 ± 1.49	7.01 ± 1.81	–2.265	0.026*
CRE (µmol/L)	79.59 ± 13.43	83.26 ± 14.49	–1.288	0.198
UA (µmol/L)	351.83 ± 88.42	334.60 ± 87.58	–0.968	0.333
FBG (mmol/L)	6.05 ± 1.43	5.90 ± 1.30	–0.636	0.525
HCY (µmol/L)	14.34 ± 4.05	16.22 ± 4.01	–2.504	0.012*
Tau (ng/L)	167.66 ± 13.72	200.86 ± 17.89	–9.584	<0.001*
Aβ (ng/L)	152.78 ± 5.28	169.97 ± 13.41	–7.017	<0.001*
SOD (U/mL)	55.78 ± 14.68	53.59 ± 9.94	–1.634	0.102
MDA (µmol/L)	2.69 ± 0.39	3.67 ± 0.52	–9.839	<0.001*
TNF-α (ng/L)	73.93 ± 7.86	85.04 ± 13.30	–4.657	<0.001*
IL-6 (ng/L)	128.75 ± 22.45	161.39 ± 19.55	–5.789	<0.001*
VILIP-1 (pg/mL)	491.13 ± 40.16	642.75 ± 98.55	–6.610	<0.001*
MiR-34c	2.78 ± 0.14	2.39 ± 0.25	8.578	<0.001*

TBIL, total bilirubin; ALT, alanine aminotransferase; AST, aspartate aminotransferase; TC, total cholesterol; TG, triglycerides; LDL-C, low-density lipoprotein cholesterol; UREA, urea nitrogen; CRE, creatinine; UA, uric acid; FBG, fasting blood glucose; HCY, homocysteine; Tau, Tau protein; Aβ, β-amyloid; SOD, superoxide dismutase; MDA, malondialdehyde; TNF-α, tumor necrosis factor-α; IL-6, interleukin-6; VILIP-1, Visinin-like protein 1; MiR-34c, microRNA-34c. *p < 0.05.

### 3.6 Correlation Between Anxiety Levels and Serum Biomarkers Within Groups

Within-group correlation analysis showed that anxiety levels correlated negatively with MiR-34c (Group A: r = –0.456, *p* = 0.003; Group B: r = –0.523, *p* < 0.001), and positively with Tau (Group A: r = 0.398, *p* = 0.010; Group B: r = 0.445, *p* = 0.002), Aβ (Group A: r = 0.367, *p* = 0.018; Group B: r = 0.412, *p* = 0.005), MDA (Group A: r = 0.423, *p* = 0.006; Group B: r = 0.478, *p* = 0.001), IL-6 (Group A: r = 0.345, *p* = 0.027; Group B: r = 0.389, *p* = 0.008), and VILIP-1 (Group A: r = 0.412, *p* = 0.007; Group B: r = 0.456, *p* = 0.002). After Bonferroni correction (α = 0.002), only MDA and MiR-34c in Group B remained significant (Table 5). Spearman correlation coefficients were used for this analysis because several biomarker distributions did not satisfy the normality assumption based on the Shapiro-Wilk test.

**Table 5.** **Within-group correlation analysis between blood biochemistry/serum biomarker levels and anxiety levels (Spearman’s correlation coefficients)**.

Project		LYM	UREA	Tau	Aβ	MDA	HCY	TNF-α	IL-6	VILIP-1	MiR-34c
Group A HAMA											
	r	–0.298	0.156	0.398	0.367	0.423	0.234	0.289	0.345	0.412	–0.456
	*p*	0.058	0.329	0.010*	0.018*	0.006	0.140	0.067	0.027*	0.007	0.003
Group B HAMA											
	r	–0.234	0.167	0.445	0.412	0.478	0.298	0.334	0.389	0.456	–0.523
	*p*	0.125	0.272	0.002	0.005*	0.001†	0.047*	0.024*	0.008*	0.002	0.000†

†Significant after Bonferroni correction (*p* < 0.002). *Significant at *p* < 0.05.

### 3.7 Multivariate Analysis

Multivariate logistic regression analysis, adjusting for age, education, smoking, and comorbidities, identified the following factors independently associated with anxiety disorders: Tau (odds ratio [OR] = 1.15, 95% CI: 1.08–1.23, *p* < 0.001), MDA (OR = 3.45, 95% CI: 1.87–6.37, *p* < 0.001), VILIP-1 (OR = 1.01, 95% CI: 1.005–1.015, *p* < 0.001), and MiR-34c (OR = 0.12, 95% CI: 0.04–0.36, *p* < 0.001). Collinearity diagnostics revealed that the VIF for the included variables was generally below 5 (range: 1.2–4.8), although moderate intercorrelation was observed between Tau and Aβ (r = 0.68) and between MDA and IL-6 (r = 0.61). The biomarker intercorrelation matrix is presented in the **Supplementary Material**. Notably, the OR for Tau (1.15 per unit increase) and VILIP-1 (1.01 per pg/mL increase) approached 1 after adjustment, suggesting that much of their univariate discriminative ability may be attributable to confounding by age, comorbidities, or intercorrelation with other biomarkers. In contrast, MiR-34c (OR = 0.12) and MDA (OR = 3.45) retained stronger independent associations with anxiety status, suggesting that these may represent more robust markers of anxiety-related biological processes.

### 3.8 ROC Curve Analysis of Serum Biomarkers for Anxiety Disorder Discrimination

**Fig. 2.**
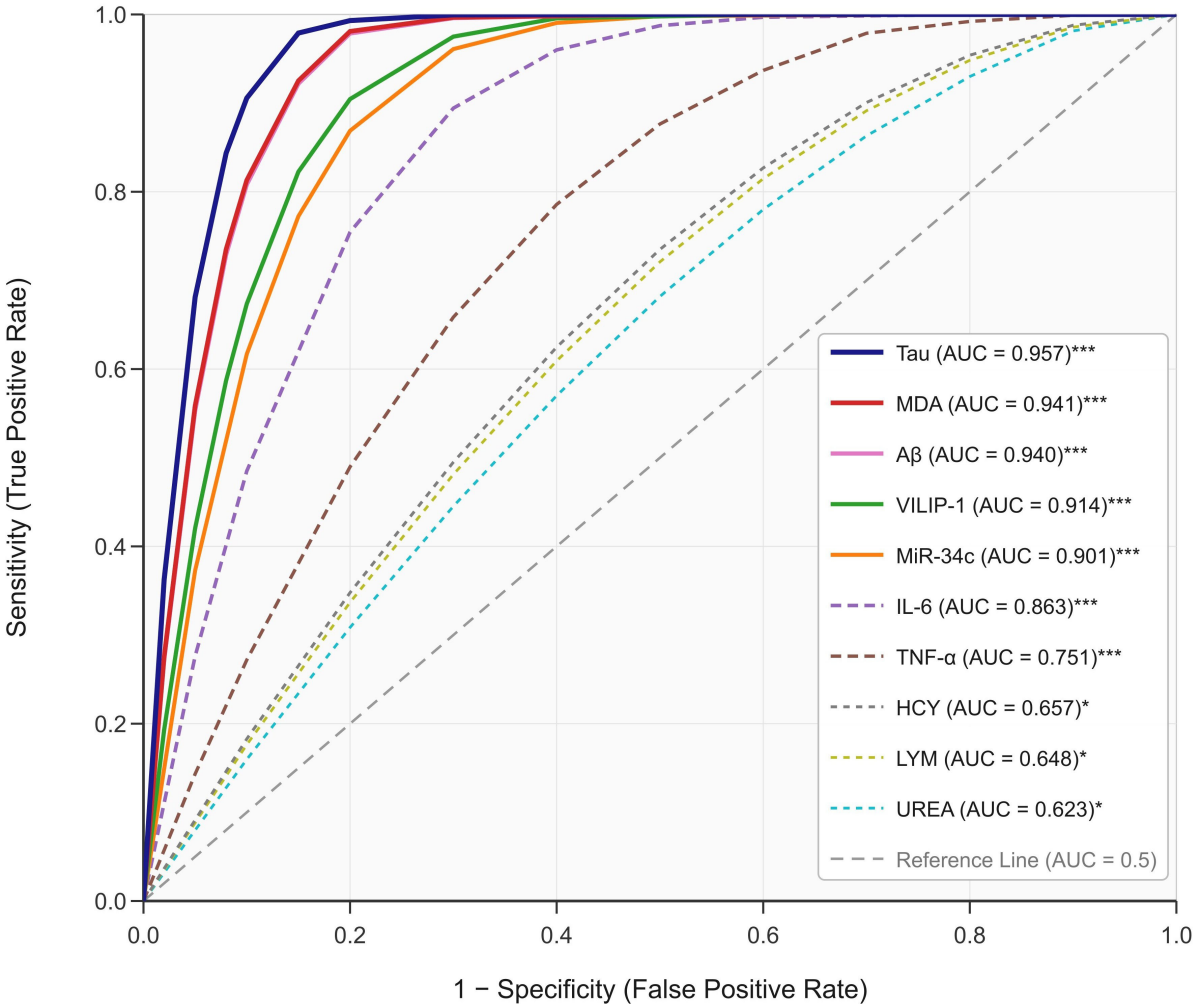
**ROC curve of serum biomarkers for discriminating anxiety disorder status**. Receiver operating characteristic curves are shown for ten serum biomarkers. The diagonal dashed reference line represents chance discrimination (AUC = 0.5). Solid lines denote biomarkers with AUC >0.90; medium-dashed lines denote AUC 0.70–0.90; short-dashed lines denote AUC <0.70. Tau protein demonstrates the highest univariate discrimination (AUC = 0.957, 95% CI: 0.921–0.993, sensitivity = 91.1%, specificity = 85.4%), followed by MDA (AUC = 0.941, 95% CI: 0.896–0.987, sensitivity = 88.9%, specificity = 87.8%) and VILIP-1 (AUC = 0.914, 95% CI: 0.858–0.970, sensitivity = 84.4%, specificity = 82.9%). ***p < 0.001; *p < 0.05. Optimal cut-off points were determined using Youden’s index. Note: Univariate AUC values do not account for confounding variables; interpretation should consider baseline group imbalances.

ROC analysis was used to evaluate the discriminative performance of serum biomarkers in differentiating patients with CI with and without comorbid anxiety disorders. ROC analysis revealed that LYM, UREA, Tau, Aβ, MDA, HCY, TNF-α, IL-6, VILIP-1, and MiR-34c had AUC values of 0.648, 0.623, 0.957, 0.940, 0.941, 0.657, 0.751, 0.863, 0.914, and 0.901, respectively, indicating their discriminative ability for anxiety status. The markers with the highest discriminative ability were Tau (AUC = 0.957, 95% CI: 0.921–0.993, sensitivity = 91.1%, specificity = 85.4%), MDA (AUC = 0.941, 95% CI: 0.896–0.987, sensitivity = 88.9%, specificity = 87.8%), and VILIP-1 (AUC = 0.914, 95% CI: 0.858–0.970, sensitivity = 84.4%, specificity = 82.9%) (Fig. 2). However, as noted above, these univariate AUC values do not account for confounding factors, and substantial baseline differences between groups may inflate the reported discriminative performance. The discrepancy between Tau’s high univariate AUC and its modest adjusted OR in the multivariate model underscores this concern.

## 4. Discussion

Understanding anxiety symptoms in elderly patients with cognitive impairment requires recognizing that both conditions often reflect shared neurobiological processes rather than representing distinct entities [13, 14]. In the context of neurodegenerative changes affecting elderly individuals, anxiety symptoms frequently emerge as neuropsychiatric manifestations of the same pathological mechanisms that drive cognitive decline.

The prefrontal-limbic circuits governing both cognitive processing and emotional regulation show overlapping dysfunctions in CI and anxiety disorders [15, 17]. A related diagnostic consideration is that the HAMA, while supplemented by DSM-5–based clinical interviews in this study, was originally developed for populations without significant cognitive impairments. In elderly patients with CI, somatic symptoms common to both conditions, such as sleep disturbance, fatigue, and concentration difficulties, may overlap, complicating the distinction between primary anxiety and anxiety arising as a behavioral and BPSD [13]. Although our exclusion of patients with pre-existing psychiatric histories was designed to reduce this ambiguity, the cross-sectional design cannot establish a temporal sequence between anxiety and cognitive decline. This diagnostic uncertainty should be considered when interpreting the associations reported below. When neurodegenerative processes affect these integrated networks, symptoms can manifest across both cognitive and emotional domains, explaining why anxiety symptoms are particularly prevalent in elderly CI patients and why our study found that Group B exhibited more severe deficits in multiple cognitive domains.

Our findings revealed that patients with CI and comorbid anxiety demonstrated significantly worse performance across most MOCA domains, with attention and delayed recall demonstrating the strongest discriminative ability (AUC >0.74). These cognitive domains are particularly vulnerable because they depend on prefrontal-hippocampal circuits, which are highly susceptible to stress-induced dysfunction and neurodegeneration [17]. The associations between these cognitive measures and anxiety severity, even within individual groups, support a dimensional relationship in which cognitive performance and emotional regulation exist on interconnected continua rather than as separate functions.

An important interpretative consideration is the possibility of bidirectional measurement bias. Anxiety can impair attentional resources, working memory capacity, and executive function, potentially resulting in lower MOCA scores that do not solely reflect underlying neurodegenerative pathology. This phenomenon has been well documented in the cognitive psychology literature, where state anxiety has been shown to consume working memory resources and disrupt prefrontal cortical efficiency. Consequently, the observed association between lower cognitive scores and higher anxiety severity may be partially attributable to the acute effects of anxiety on test performance rather than exclusively reflecting shared neurodegeneration. This bidirectional relationship represents a fundamental limitation of cross-sectional assessments and can only be disentangled through longitudinal designs with repeated cognitive evaluations under varying anxiety states.

The biomarker findings provide potential mechanistic insights into the shared pathophysiology underlying CI-anxiety comorbidity, although these interpretations remain speculative given the cross-sectional design. The inflammatory hypothesis of neuropsychiatric disorders offers a framework for understanding these observations [19, 20]. Elevated IL-6 and TNF-α levels in the anxiety group reflect chronic inflammatory activation, which has been shown in prior studies to impair cognition through microglial activation, synaptic dysfunction, and interference with neurotransmitter metabolism [22, 23]. IL-6 specifically disrupts serotonin synthesis by increasing indoleamine 2,3-dioxygenase activity, creating a mechanistic link between inflammation and mood dysregulation. Our findings are consistent with prior reports linking elevated peripheral inflammatory markers to neuropsychiatric symptoms in elderly populations, including a meta-analysis by Custodero *et al*. [10] demonstrating associations between inflammatory biomarkers and cognitive impairment with neuropsychiatric features. However, although significant, the within-group correlations between inflammatory markers and anxiety severity were modest in magnitude, and the causal direction of this relationship could not be determined from cross-sectional data.

The significantly elevated Tau and Aβ levels in patients with anxiety suggest an association with accelerated neurodegeneration processes. While traditionally associated with Alzheimer’s pathology, these proteins also reflect acute neuronal stress and injury [21]. However, the multivariate analysis revealed that the adjusted OR for Tau was relatively modest (1.15 per unit increase), and the discrepancy between Tau’s high univariate AUC (0.957) and its attenuated effect in the adjusted model suggests that much of Tau’s discriminative ability in univariate analysis may reflect confounding by age, comorbidities, or intercorrelation with other biomarkers rather than a direct and independent association with anxiety status. This finding underscores the importance of multivariate adjustment when interpreting biomarker associations in observational studies with baseline imbalance.

Oxidative stress represents another convergent pathway, as evidenced by markedly elevated MDA levels in the anxiety group. This finding reflects compromised cellular antioxidant defenses and increased lipid peroxidation [24, 25]. MDA retained a strong independent association with anxiety status in the multivariate model (OR = 3.45), suggesting that oxidative stress may represent a more robust biological correlate of anxiety in this population, although confirmatory longitudinal studies are needed.

VILIP-1 emerged as one of our strongest discriminators (AUC = 0.914), consistent with its role as a sensitive marker of neuronal calcium dysregulation and cellular stress [26, 27]. Its elevation suggests widespread neuronal vulnerability extending beyond traditional cognitive networks to encompass the emotional processing regions. Similarly, the significant reduction in MiR-34c levels in patients with anxiety may represent impaired stress resilience mechanisms and reduced neuroprotective capacity [28]. MiR-34c demonstrated the strongest independent association with anxiety in the multivariate model (OR = 0.12), suggesting that it may be a particularly informative molecular marker worthy of further investigation in prospective studies. Our findings are broadly consistent with emerging evidence implicating miRNA dysregulation in neuropsychiatric conditions, as reviewed by Albano *et al*. [28], although direct comparisons are limited by differences in study populations and biomarker panels.

Our multivariate analysis, incorporating the Bonferroni correction for multiple testing, identified independent biomarker factors after controlling for demographic and clinical confounders. The persistence of significant associations for the oxidative stress indicator MDA and regulatory microRNA MiR-34c suggests that these may represent informative biological correlates rather than secondary effects, although validation in independent cohorts is essential. It must be acknowledged, however, that the pronounced baseline imbalances between the study groups—particularly the nearly threefold difference in the proportion of patients aged ≥70 years and the more than twofold difference in diabetes prevalence—raise the possibility that multivariable regression may not fully resolve this degree of structural non-comparability. When groups differ so markedly across multiple confounding dimensions simultaneously, the functional form assumptions underpinning parametric adjustment become more tenuous, and residual confounding from both measured and unmeasured variables remains a plausible alternative explanation for the observed associations. Accordingly, all biomarker associations identified in this study should be interpreted as exploratory findings that require confirmation in studies employing either design-based solutions (such as frequency-matched enrollment or propensity score methods) or prospective longitudinal designs with more balanced comparison groups. Similarly, the ROC/AUC findings reported herein represent hypothesis-generating assessments of discrimination performance within this specific cohort and should not be interpreted as evidence of clinically actionable diagnostic or predictive utility in the absence of external validation.

Regarding the generalizability of these findings, the restriction to male participants requires careful consideration. Sex differences in anxiety disorders and cognitive impairment are well documented, encompassing hormonal influences (e.g., estrogen-mediated neuroprotection), differential inflammatory and oxidative stress profiles, and variations in hypothalamic-pituitary-adrenal axis reactivity (HPA). These factors may substantially modify the biomarker associations observed in the present study, and our results should not be extrapolated to the female population. Beyond the sex restriction, the derivation of our study population from a single military veteran rehabilitation facility introduces a further dimension of selection that merits explicit consideration. Retired military veterans may carry a distinctive burden of cumulative psychological stress—including potential combat-related trauma exposure—that could independently modulate inflammatory, oxidative stress, and neurodegeneration biomarker profiles. Moreover, this population may differ from community-dwelling elderly individuals in terms of healthcare access patterns, medication use, lifestyle factors, and the nature and severity of chronic medical comorbidities. These selection-related characteristics represent sources of potential bias that are not captured by the covariates included in our regression model, and they further limit the extent to which the present findings can be extrapolated to the broader elderly CI population. Multicenter studies incorporating both sexes and adequate power for sex-stratified analyses are a critical priority for future research.

Future investigations would also benefit from employing more comprehensive neuropsychological batteries beyond the MOCA screening tool. While the MOCA is well-validated and appropriate for the clinical screening context of this study, detailed assessments of episodic memory (e.g., the Rey Auditory Verbal Learning Test), executive function (e.g., the Trail Making Test), language (e.g., the Boston Naming Test), and processing speed would provide more granular cognitive profiling and potentially improve the specificity of cognitive biomarker assessment approaches.

The clinical implications include the potential future development of biomarker-based screening approaches for anxiety identification in elderly CI populations, although the present findings are preliminary and hypothesis-generating. If validated in prospective studies with external cohorts, the identification of specific pathways (inflammation, oxidative stress, and neurodegeneration) could provide targets for therapeutic approaches, such as anti-inflammatory strategies and neuroprotective interventions.

### 4.1 Study Limitations

This study had several limitations that warrant consideration. The cross-sectional retrospective design precludes causal or temporal inference, meaning that the observed associations cannot be interpreted as predictive or directional. Although DSM-5–based clinical interviews supplemented HAMA screening, the inability to definitively distinguish primary anxiety from BPSD-related symptomatology remains an inherent limitation in this cognitively impaired population. The male-only sample from a single military veteran rehabilitation facility limits generalizability to women and non-veteran populations, given the well-documented sex differences in anxiety epidemiology, hormonal neuroprotection, inflammatory profiles, and stress reactivity. As a highly selected subgroup of retired military veterans, this population may carry distinctive psychological stress exposure histories and comorbidity profiles that limit the generalizability of these findings to the broader elderly CI population. The modest sample size, despite adequate a priori power, raises concerns regarding overfitting in the multivariate model. Pronounced baseline imbalances in age, education, and comorbidity burden between groups represent a major concern; although multivariate adjustment was performed, regression adjustment alone may not fully resolve structural non-comparability of this magnitude, and residual confounding cannot be excluded from the results. The absence of external validation, moderate intercorrelation among biomarkers (as reflected in the VIF values and correlation matrix), and potential influence of subclinical inflammation on biomarker levels further limit the robustness and generalizability of these findings. Finally, the bidirectional relationship between anxiety and cognitive test performance complicates the interpretation of cognitive-anxiety associations, as anxiety may independently impair MOCA performance beyond any shared neurodegenerative substrate.

### 4.2 Take Home Message

Anxiety symptoms in elderly men with cognitive impairment may reflect shared neurobiological processes rather than separate psychiatric disorders. MOCA attention and delayed recall domains, combined with serum biomarkers (Tau, MDA, VILIP-1, and MiR-34c), demonstrated the ability to discriminate anxiety status in this population. The inflammatory and oxidative stress pathways identified warrant further investigation as potential therapeutic targets for integrated cognitive-emotional interventions in elderly patients with cognitive impairment and comorbid anxiety. Clinicians should consider routine anxiety screening in elderly cognitive impairment patients using both cognitive assessment and biomarker profiling, pending validation in larger prospective studies. These findings are hypothesis-generating and require confirmation prior to clinical implementation. Given the single-center military veteran origin of this cohort and the pronounced baseline imbalances between groups, replication in diverse, multicenter, sex-inclusive populations with balanced comparison groups is essential before clinical translation can be considered.

## 5. Conclusion

This cross-sectional study identified associations between CI and anxiety disorders, demonstrating that MOCA scores (attention, delayed recall) and serum biomarkers (Tau, MDA, VILIP-1, and MiR-34c) exhibit discriminative ability for anxiety status in elderly men. These findings are consistent with a neurobiological model of CI-anxiety comorbidity involving inflammatory, oxidative stress, and neuronal damage pathways. However, these findings should be regarded as hypothesis-generating rather than confirmatory findings. Importantly, these results derive from a single-center cohort of retired male military veterans with pronounced baseline group imbalances, substantially limiting generalizability to the broader elderly CI population and necessitating that all reported biomarker associations be interpreted as exploratory. Limited by its cross-sectional design, use of severity screening rather than comprehensive diagnostic criteria for anxiety, inability to distinguish primary anxiety from BPSD-related symptomatology, small sample size, pronounced baseline imbalances between groups, male-only single-center focus, and absence of external validation, further prospective, longitudinal multi-center studies with comprehensive psychiatric assessment and sex-inclusive cohorts are needed to validate these findings before any clinical application can be considered.

## Availability of Data and Materials

The data presented in this study are available on reasonable request from the corresponding author.

## Supplementary Material

Supplementary material associated with this article can be found, in the online version, at https://doi.org/10.31083/RN48908.
