# Supplementary material for: Association of MOCA Cognitive Domains and Serum Biomarkers With Anxiety Disorders in Elderly Men With Cognitive Impairment: A Cross-Sectional Analysis
Source: Rev Neurol. 2026 Apr 21;81(4):48908. doi: 10.31083/RN48908 (PMC13129556; doi:10.31083/RN48908)
Supplement: Supplementary file 1 [file 1576-6578-81-4-48908-s1.zip › Supplementary Material.docx]

**Supplementary Material**

*Association of MOCA Cognitive Domains and Serum Biomarkers With Anxiety Disorders in Elderly Men With Cognitive Impairment: A Cross-Sectional Analysis*

**Supplementary Table 1.** Intercorrelation matrix of serum biomarkers (Spearman’s correlation coefficients, upper triangle; n = 86).

|  | **Tau** | **Aβ** | **MDA** | **TNF-α** | **IL-6** | **VILIP-1** | **MiR-34c** |
| --- | --- | --- | --- | --- | --- | --- | --- |
| **Tau** | 1.000 | 0.680*** | 0.483*** | 0.418*** | 0.452*** | 0.548*** | -0.523*** |
| **Aβ** | — | 1.000 | 0.437*** | 0.382*** | 0.412*** | 0.516*** | -0.468*** |
| **MDA** | — | — | 1.000 | 0.543*** | 0.610*** | 0.562*** | -0.578*** |
| **TNF-α** | — | — | — | 1.000 | 0.571*** | 0.428*** | -0.392*** |
| **IL-6** | — | — | — | — | 1.000 | 0.467*** | -0.441*** |
| **VILIP-1** | — | — | — | — | — | 1.000 | -0.512*** |
| **MiR-34c** | — | — | — | — | — | — | 1.000 |

Aβ, β-amyloid; MDA, malondialdehyde; TNF-α, tumor necrosis factor-α; IL-6, interleukin-6; VILIP-1, visinin-like protein 1; MiR-34c, microRNA-34c. Lower triangle shows — (mirror values omitted for clarity). ***p < 0.001. Correlations were computed across the full sample (N = 86).

**Supplementary Table 2.** Variance inflation factors (VIF) for variables included in the multivariate logistic regression model.

| **Variable** | **VIF** |
| --- | --- |
| Tau | 3.82 |
| Aβ | 2.94 |
| MDA | 4.15 |
| TNF-α | 2.67 |
| IL-6 | 3.41 |
| VILIP-1 | 2.89 |
| MiR-34c | 2.53 |
| Age (≥70 years) | 1.48 |
| Education (≤9 years) | 1.23 |
| Smoking | 1.35 |
| Hypertension | 1.67 |
| Diabetes | 1.54 |
| Coronary heart disease | 1.42 |
| Ischemic stroke | 1.28 |

VIF values < 5 indicate acceptable multicollinearity. All variables demonstrated VIF values within the acceptable range (1.23–4.15), consistent with the reported range of 1.2–4.8 in the main text. Moderate intercorrelation was observed between Tau and Aβ (r = 0.68) and between MDA and IL-6 (r = 0.61), as detailed in Supplementary Table S1.
